# Supplementary material for: A novel automated morphological analysis of Iba1+ microglia using a deep learning assisted model
Source: Front Cell Neurosci. 2022 Sep 15;16:944875. doi: 10.3389/fncel.2022.944875 (PMC9520629; doi:10.3389/fncel.2022.944875)
Supplement: Supplementary file 1 [file Table_1.pdf]

Supplementary Table 1 – acquisition duration data

Method	Date	Start time	Stop time	Step	Total time	Figure reference
**MATLAB**	03.29.31	10:00	12:30	Brightfield imaging	2.5hrs	Fig 1a
**Aiforia**	11.01.21	18:33	19:35	ROI annotation	1hr	Fig 1a
**MATLAB**	03.30.21	13:30	15:00	Analysis / Quantification	1.5hrs	Fig 1a
**Aiforia**	11.01.21	19:37	19:43	Analysis / Quantification	5min	Fig 1a
**MATLAB**	03.29.21	13:30	14:00	Analysis / Quantification	30min	Fig 1b
**Aiforia**	4.22.21	11:31	11:35	ROI annotation	4min	Fig 1b
**Aiforia**	4.22.21	11:37	11:37	Analysis / Quantification	<1min	Fig 1b

Supplementary Table 1. Method step-specific comparison of dataset acquisition duration. (*Method*) Identifying the use of either MATLAB or Aiforia®. **(Date)** the date on which the Step was performed using US calendar notation (mm-dd-yy). **(Start time / Stop time)** the time when each Step began and ended respectively using 24-hour clock. **(Step)** a brief description of the action performed in the process of acquiring data. **(Total time)** the duration of elapsed time between when each step began and ended. **(Figure reference)** identifies the figure in the main text for which the details are provided.
