# Supplementary figures and images for: A novel automated morphological analysis of Iba1+ microglia using a deep learning assisted model
Source: Front Cell Neurosci. 2022 Sep 15;16:944875. doi: 10.3389/fncel.2022.944875 (PMC9520629; doi:10.3389/fncel.2022.944875)

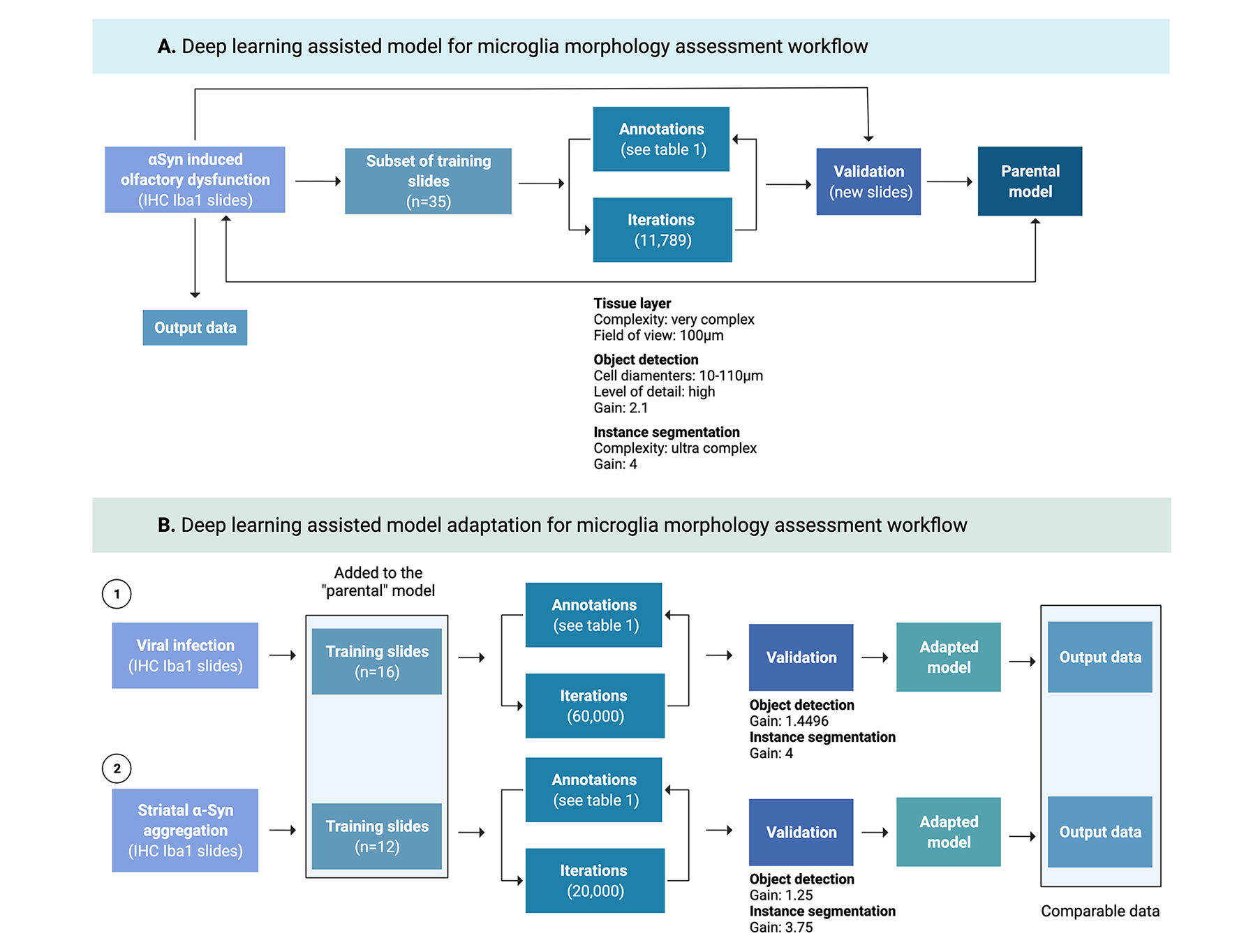

Supplement: Supplementary Figure 1 — Workflows for the development and adaptation of deep learning assisted models for microglial morphology assessment using Aiforia®. (A) Workflow for the development of microglial morphology assessment model. The “parental” model was developed with a subset of 35 IHC anti-Iba1 slides from a mouse model of αSyn induced olfactory dysfunction used for training input data (see Table 1). Details for the final round of iterations and the CNN settings by feature layer are included (network complexity, field of view, post training gains, etc.). After training, a new subset of slides (not used during the training phase) was used for validation, and cell examples were used to compare the model performance against 4–5 researchers experienced in microglial histology. (B) Workflow for the adaptation of the “parental” model to independently acquired datasets. Project-specific AIs were adapted by including additional IHC training data from mouse models of (1) viral infection and (2) striatal αSyn aggregation, to the “parental” model training data. Pre-training parameters were the same as in the “parental” model, and post-training parameters were optimized for object detection and instance segmentation as shown for each of the adapted models. New slides (not included in the training data) were used during validation against researchers experienced in microglial histology. Validated models were released and used for the quantification of microglial morphology from the Iba1 stain slides from all three mouse models. By validating each model to the same standard, analysis results were comparable (created with BioRender.com). [file Image_1.TIF]

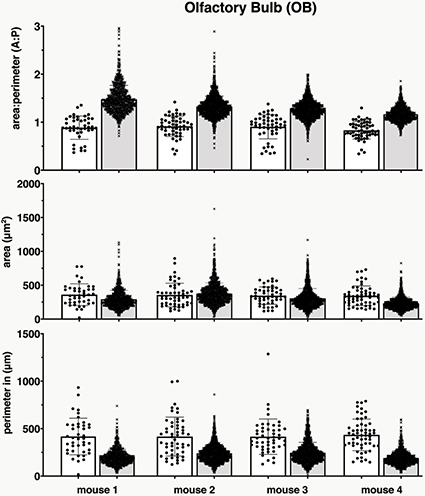

Supplement: Supplementary Figure 2 — Comparison of area and perimeter values between MATLAB and Aiforia®. Olfactory bulb Iba1 + microglia area/perimeter ratio, area, and perimeter values across 4 mice between methods, suggesting differences in area/perimeter ratio values are possibly the result of differences in perimeter value quantifications. Each histogram includes data from 1 mouse, each marker represents the values for one cell. Data collected using MATLAB represented in white bars, data collected using Aiforia® in gray. [file Image_2.JPEG]
