# Supplementary material for: A novel automated morphological analysis of Iba1+ microglia using a deep learning assisted model
Source: Front Cell Neurosci. 2022 Sep 15;16:944875. doi: 10.3389/fncel.2022.944875 (PMC9520629; doi:10.3389/fncel.2022.944875)
Supplement: Supplementary file 4 [file Data_Sheet_1.ZIP › supplemental post processing files/VAI Iba1 post processing.v2.nb.nb.html]

VAI Iba1 postprocessing of AIfria details output


Code 

- Show All Code
- Hide All Code
- Download Rmd

# VAI Iba1 postprocessing of AIfria details output

This is an R Markdown Notebook designed to extract data features from the AIforia Details analysis output, without separating results by image. Not this notbook does not work with the summary output, or with outputs separated by image at the time of export fromn the AIforia platform .

To executing this chunk of code click the *Run* button within the chunk or by placing your cursor inside it and pressing *Cmd+Shift+Enter*.

Install packages


```
install.packages("dplyr")
install.packages("svDialogs")
```


1 load the data


```
library(dplyr)
RawAIforia_details_output <- readxl::read_xlsx(file.choose())
```


2 load the treatment info (double check the image names match the AIforia details output exactly)


```
TreatmentInfoKey <- readxl::read_xlsx(file.choose())
```


3 load the region of interest key (double check that all ROI’ names for the same brain region are encoded the same)


```
ROIKey <- readxl::read_xlsx(file.choose())
```


4 run the post-processing script


```
roi_tissue_areas <- RawAIforia_details_output %>%
  filter(`Class label` == 'Tissue') %>%
  group_by(ROI) %>%
  summarise(roi_tissue_area_um2 = sum(`Area (μm²)`))

# Area of parent tissue
parent_tissue_areas <- RawAIforia_details_output %>%
  filter(`Class label` == 'Tissue') %>%
  group_by(`Area/object ID`) %>%
  summarise(parent_tissue_area_um2 = sum(`Area (μm²)`))
parent_tissue_areas$parent_tissue_area_mm2 <- parent_tissue_areas$parent_tissue_area_um2 * 0.000001

roi_letter_pattern <- ROIKey$`letters found in ROI label used to code brain region` %>% paste(collapse = '|')

# Combine treatment info with data
RawAIforia_details_output_PlusTreatmentInfo <- RawAIforia_details_output %>%
  mutate(
    area_to_circumference = `Area (μm²)` / `Circumference (µm)`
  ) %>%
  left_join(
    roi_tissue_areas,
    by = 'ROI'
  ) %>%
  left_join(
    parent_tissue_areas,
    by = c('Parent area ID' = 'Area/object ID')
  ) %>%
  # Add brain region with roi label key
  mutate(
    roi_letters = regexpr(roi_letter_pattern, ROI) %>% regmatches(ROI, .)
  ) %>%
  left_join(
    select(ROIKey, `letters found in ROI label used to code brain region`, `Brain region`),
    by = c('roi_letters' = 'letters found in ROI label used to code brain region')
  ) %>%
  # Add treatment info with image name
  left_join(
    TreatmentInfoKey,
    by = c('Image' = 'image name')
  )

#start ptII
SummaryByROI <- RawAIforia_details_output_PlusTreatmentInfo %>%
  #filter(`Class label` != 'Tissue') %>%
  group_by(Image, ROI, `Class label`, `Brain region`, `treatment info`, `parent_tissue_area_mm2` ) %>%
  mutate(Count = n()) %>%
  #filter(Count >100) %>% #this is a little arbitrary but removes failed ROIs
  group_by(Image, ROI, `Class label`, `Brain region`, `treatment info`, `parent_tissue_area_mm2`, `Count` ) %>%
  
  summarise( 
    mean_object_diameter_um = mean(`Object diameter (μm)`, na.rm = TRUE),
    mean_area_to_circumference = mean(`area_to_circumference`, na.rm = TRUE),
    mean_area_um = mean(`Area (μm²)`, na.rm = TRUE),
    mean_circumference_um = mean(`Circumference (µm)`, na.rm = TRUE),
    mean_length_um = mean(`Length (µm)`, na.rm = TRUE),
    mean_b = mean(`B`, na.rm = TRUE),
    mean_g = mean(`G`, na.rm = TRUE),
    mean_r = mean(`R`, na.rm = TRUE),
  )

openxlsx::write.xlsx(SummaryByROI,
                     file = "SummaryByROI.xlsx")
#pt.III
#Brain region1
library(svDialogs)
BrainRegion1_user.input <- dlgInput("Enter the label used for brain region 1", Sys.info()["user"])$res

BR1 <- SummaryByROI %>% 
  filter(`Brain region` %in% c(BrainRegion1_user.input))%>%
  group_by(Image, `treatment info`,  ) %>%
  
  summarise( 
    total_Count = sum(Count, na.rm = TRUE),
    total_Parent_Tissue_Area_mm2 = sum(`parent_tissue_area_mm2`, na.rm = TRUE),
    mean_Parent_Tissue_Area_mm2 = mean(`parent_tissue_area_mm2`, na.rm = TRUE),
    mean_Object_Diameter_um = mean(`mean_object_diameter_um`, na.rm = TRUE),
    mean_Area_to_Circumference = mean(`mean_area_to_circumference`, na.rm = TRUE),
    mean_Area_um = mean(`mean_area_um`, na.rm = TRUE),
    mean_Circumference_um = mean(`mean_circumference_um`, na.rm = TRUE),
    mean_Length_um = mean(`mean_length_um`, na.rm = TRUE),
    mean_B = mean(`mean_b`, na.rm = TRUE),
    mean_G = mean(`mean_g`, na.rm = TRUE),
    mean_R = mean(`mean_r`, na.rm = TRUE),
  )

openxlsx::write.xlsx(BR1,
                     file = paste0(BrainRegion1_user.input,'.xlsx'))

#Brain region2
library(svDialogs)
BrainRegion2_user.input <- dlgInput("Enter the label used for brain region 2", Sys.info()["user"])$res

BR2 <- SummaryByROI %>% 
  filter(`Brain region` %in% c(BrainRegion2_user.input))%>%
  group_by(Image, `treatment info`,  ) %>%
  
  summarise( 
    total_Count = sum(Count, na.rm = TRUE),
    total_Parent_Tissue_Area_mm2 = sum(`parent_tissue_area_mm2`, na.rm = TRUE),
    mean_Parent_Tissue_Area_mm2 = mean(`parent_tissue_area_mm2`, na.rm = TRUE),
    mean_Object_Diameter_um = mean(`mean_object_diameter_um`, na.rm = TRUE),
    mean_Area_to_Circumference = mean(`mean_area_to_circumference`, na.rm = TRUE),
    mean_Area_um = mean(`mean_area_um`, na.rm = TRUE),
    mean_Circumference_um = mean(`mean_circumference_um`, na.rm = TRUE),
    mean_Length_um = mean(`mean_length_um`, na.rm = TRUE),
    mean_B = mean(`mean_b`, na.rm = TRUE),
    mean_G = mean(`mean_g`, na.rm = TRUE),
    mean_R = mean(`mean_r`, na.rm = TRUE),
  )

openxlsx::write.xlsx(BR2,
                     file = paste0(BrainRegion2_user.input,'.xlsx'))

#Brain region3
library(svDialogs)
BrainRegion3_user.input <- dlgInput("Enter the label used for brain region 3", Sys.info()["user"])$res

BR3 <- SummaryByROI %>% 
  filter(`Brain region` %in% c(BrainRegion3_user.input))%>%
  group_by(Image, `treatment info`,  ) %>%
  
  summarise( 
    total_Count = sum(Count, na.rm = TRUE),
    total_Parent_Tissue_Area_mm2 = sum(`parent_tissue_area_mm2`, na.rm = TRUE),
    mean_Parent_Tissue_Area_mm2 = mean(`parent_tissue_area_mm2`, na.rm = TRUE),
    mean_Object_Diameter_um = mean(`mean_object_diameter_um`, na.rm = TRUE),
    mean_Area_to_Circumference = mean(`mean_area_to_circumference`, na.rm = TRUE),
    mean_Area_um = mean(`mean_area_um`, na.rm = TRUE),
    mean_Circumference_um = mean(`mean_circumference_um`, na.rm = TRUE),
    mean_Length_um = mean(`mean_length_um`, na.rm = TRUE),
    mean_B = mean(`mean_b`, na.rm = TRUE),
    mean_G = mean(`mean_g`, na.rm = TRUE),
    mean_R = mean(`mean_r`, na.rm = TRUE),
  )

openxlsx::write.xlsx(BR3,
                     file = paste0(BrainRegion3_user.input,'.xlsx'))

#Brain region4
library(svDialogs)
BrainRegion4_user.input <- dlgInput("Enter the label used for brain region 4", Sys.info()["user"])$res

BR4 <- SummaryByROI %>% 
  filter(`Brain region` %in% c(BrainRegion4_user.input))%>%
  group_by(Image, `treatment info`,  ) %>%
  
  summarise( 
    total_Count = sum(Count, na.rm = TRUE),
    total_Parent_Tissue_Area_mm2 = sum(`parent_tissue_area_mm2`, na.rm = TRUE),
    mean_Parent_Tissue_Area_mm2 = mean(`parent_tissue_area_mm2`, na.rm = TRUE),
    mean_Object_Diameter_um = mean(`mean_object_diameter_um`, na.rm = TRUE),
    mean_Area_to_Circumference = mean(`mean_area_to_circumference`, na.rm = TRUE),
    mean_Area_um = mean(`mean_area_um`, na.rm = TRUE),
    mean_Circumference_um = mean(`mean_circumference_um`, na.rm = TRUE),
    mean_Length_um = mean(`mean_length_um`, na.rm = TRUE),
    mean_B = mean(`mean_b`, na.rm = TRUE),
    mean_G = mean(`mean_g`, na.rm = TRUE),
    mean_R = mean(`mean_r`, na.rm = TRUE),
  )

openxlsx::write.xlsx(BR4,
                     file = paste0(BrainRegion4_user.input,'.xlsx'))

#Brain region5
library(svDialogs)
BrainRegion5_user.input <- dlgInput("Enter the label used for brain region 5", Sys.info()["user"])$res

BR5 <- SummaryByROI %>% 
  filter(`Brain region` %in% c(BrainRegion5_user.input))%>%
  group_by(Image, `treatment info`,  ) %>%
  
  summarise( 
    total_Count = sum(Count, na.rm = TRUE),
    total_Parent_Tissue_Area_mm2 = sum(`parent_tissue_area_mm2`, na.rm = TRUE),
    mean_Parent_Tissue_Area_mm2 = mean(`parent_tissue_area_mm2`, na.rm = TRUE),
    mean_Object_Diameter_um = mean(`mean_object_diameter_um`, na.rm = TRUE),
    mean_Area_to_Circumference = mean(`mean_area_to_circumference`, na.rm = TRUE),
    mean_Area_um = mean(`mean_area_um`, na.rm = TRUE),
    mean_Circumference_um = mean(`mean_circumference_um`, na.rm = TRUE),
    mean_Length_um = mean(`mean_length_um`, na.rm = TRUE),
    mean_B = mean(`mean_b`, na.rm = TRUE),
    mean_G = mean(`mean_g`, na.rm = TRUE),
    mean_R = mean(`mean_r`, na.rm = TRUE),
  )

openxlsx::write.xlsx(BR5,
                     file = paste0(BrainRegion5_user.input,'.xlsx'))

#Brain region6
library(svDialogs)
BrainRegion6_user.input <- dlgInput("Enter the label used for brain region 6", Sys.info()["user"])$res

BR6 <- SummaryByROI %>% 
  filter(`Brain region` %in% c(BrainRegion6_user.input))%>%
  group_by(Image, `treatment info`,  ) %>%
  
  summarise( 
    total_Count = sum(Count, na.rm = TRUE),
    total_Parent_Tissue_Area_mm2 = sum(`parent_tissue_area_mm2`, na.rm = TRUE),
    mean_Parent_Tissue_Area_mm2 = mean(`parent_tissue_area_mm2`, na.rm = TRUE),
    mean_Object_Diameter_um = mean(`mean_object_diameter_um`, na.rm = TRUE),
    mean_Area_to_Circumference = mean(`mean_area_to_circumference`, na.rm = TRUE),
    mean_Area_um = mean(`mean_area_um`, na.rm = TRUE),
    mean_Circumference_um = mean(`mean_circumference_um`, na.rm = TRUE),
    mean_Length_um = mean(`mean_length_um`, na.rm = TRUE),
    mean_B = mean(`mean_b`, na.rm = TRUE),
    mean_G = mean(`mean_g`, na.rm = TRUE),
    mean_R = mean(`mean_r`, na.rm = TRUE),
  )

openxlsx::write.xlsx(BR6,
                     file = paste0(BrainRegion6_user.input,'.xlsx'))

#Brain region7
library(svDialogs)
BrainRegion7_user.input <- dlgInput("Enter the label used for brain region 7", Sys.info()["user"])$res

BR7 <- SummaryByROI %>% 
  filter(`Brain region` %in% c(BrainRegion7_user.input))%>%
  group_by(Image, `treatment info`,  ) %>%
  
  summarise( 
    total_Count = sum(Count, na.rm = TRUE),
    total_Parent_Tissue_Area_mm2 = sum(`parent_tissue_area_mm2`, na.rm = TRUE),
    mean_Parent_Tissue_Area_mm2 = mean(`parent_tissue_area_mm2`, na.rm = TRUE),
    mean_Object_Diameter_um = mean(`mean_object_diameter_um`, na.rm = TRUE),
    mean_Area_to_Circumference = mean(`mean_area_to_circumference`, na.rm = TRUE),
    mean_Area_um = mean(`mean_area_um`, na.rm = TRUE),
    mean_Circumference_um = mean(`mean_circumference_um`, na.rm = TRUE),
    mean_Length_um = mean(`mean_length_um`, na.rm = TRUE),
    mean_B = mean(`mean_b`, na.rm = TRUE),
    mean_G = mean(`mean_g`, na.rm = TRUE),
    mean_R = mean(`mean_r`, na.rm = TRUE),
  )

openxlsx::write.xlsx(BR7,
                     file = paste0(BrainRegion7_user.input,'.xlsx'))

#Brain region8
library(svDialogs)
BrainRegion8_user.input <- dlgInput("Enter the label used for brain region 8", Sys.info()["user"])$res

BR8 <- SummaryByROI %>% 
  filter(`Brain region` %in% c(BrainRegion8_user.input))%>%
  group_by(Image, `treatment info`,  ) %>%
  
  summarise( 
    total_Count = sum(Count, na.rm = TRUE),
    total_Parent_Tissue_Area_mm2 = sum(`parent_tissue_area_mm2`, na.rm = TRUE),
    mean_Parent_Tissue_Area_mm2 = mean(`parent_tissue_area_mm2`, na.rm = TRUE),
    mean_Object_Diameter_um = mean(`mean_object_diameter_um`, na.rm = TRUE),
    mean_Area_to_Circumference = mean(`mean_area_to_circumference`, na.rm = TRUE),
    mean_Area_um = mean(`mean_area_um`, na.rm = TRUE),
    mean_Circumference_um = mean(`mean_circumference_um`, na.rm = TRUE),
    mean_Length_um = mean(`mean_length_um`, na.rm = TRUE),
    mean_B = mean(`mean_b`, na.rm = TRUE),
    mean_G = mean(`mean_g`, na.rm = TRUE),
    mean_R = mean(`mean_r`, na.rm = TRUE),
  )

openxlsx::write.xlsx(BR8,
                     file = paste0(BrainRegion8_user.input,'.xlsx'))
```


```
ROIKey <- readxl::read_xlsx(file.choose())
```


LS0tCnRpdGxlOiAiVkFJIEliYTEgcG9zdHByb2Nlc3Npbmcgb2YgQUlmcmlhIGRldGFpbHMgb3V0cHV0IgpvdXRwdXQ6IGh0bWxfbm90ZWJvb2sKLS0tCgpUaGlzIGlzIGFuIFtSIE1hcmtkb3duXShodHRwOi8vcm1hcmtkb3duLnJzdHVkaW8uY29tKSBOb3RlYm9vayBkZXNpZ25lZCB0byBleHRyYWN0IGRhdGEgZmVhdHVyZXMgZnJvbSB0aGUgQUlmb3JpYSBEZXRhaWxzIGFuYWx5c2lzIG91dHB1dCwgd2l0aG91dCBzZXBhcmF0aW5nIHJlc3VsdHMgYnkgaW1hZ2UuIE5vdCB0aGlzIG5vdGJvb2sgZG9lcyBub3Qgd29yayB3aXRoIHRoZSBzdW1tYXJ5IG91dHB1dCwgb3Igd2l0aCBvdXRwdXRzIHNlcGFyYXRlZCBieSBpbWFnZSBhdCB0aGUgdGltZSBvZiBleHBvcnQgZnJvbW4gdGhlIEFJZm9yaWEgcGxhdGZvcm0gLgoKVG8gZXhlY3V0aW5nIHRoaXMgY2h1bmsgb2YgY29kZSBjbGljayB0aGUgKlJ1biogYnV0dG9uIHdpdGhpbiB0aGUgY2h1bmsgb3IgYnkgcGxhY2luZyB5b3VyIGN1cnNvciBpbnNpZGUgaXQgYW5kIHByZXNzaW5nICpDbWQrU2hpZnQrRW50ZXIqLiAKCkluc3RhbGwgcGFja2FnZXMgCmBgYHtyfQppbnN0YWxsLnBhY2thZ2VzKCJkcGx5ciIpCmluc3RhbGwucGFja2FnZXMoInN2RGlhbG9ncyIpCgpgYGAgCgoxIGxvYWQgdGhlIGRhdGEKYGBge3J9CmxpYnJhcnkoZHBseXIpClJhd0FJZm9yaWFfZGV0YWlsc19vdXRwdXQgPC0gcmVhZHhsOjpyZWFkX3hsc3goZmlsZS5jaG9vc2UoKSkKYGBgCjIgbG9hZCB0aGUgdHJlYXRtZW50IGluZm8gKGRvdWJsZSBjaGVjayB0aGUgaW1hZ2UgbmFtZXMgbWF0Y2ggdGhlIEFJZm9yaWEgZGV0YWlscyBvdXRwdXQgZXhhY3RseSkKYGBge3J9ClRyZWF0bWVudEluZm9LZXkgPC0gcmVhZHhsOjpyZWFkX3hsc3goZmlsZS5jaG9vc2UoKSkKYGBgCgozIGxvYWQgdGhlIHJlZ2lvbiBvZiBpbnRlcmVzdCBrZXkgKGRvdWJsZSBjaGVjayB0aGF0IGFsbCBST0knIG5hbWVzIGZvciB0aGUgc2FtZSBicmFpbiByZWdpb24gYXJlIGVuY29kZWQgdGhlIHNhbWUpCmBgYHtyfQpST0lLZXkgPC0gcmVhZHhsOjpyZWFkX3hsc3goZmlsZS5jaG9vc2UoKSkKYGBgCgo0IHJ1biB0aGUgcG9zdC1wcm9jZXNzaW5nIHNjcmlwdApgYGB7cn0KCnJvaV90aXNzdWVfYXJlYXMgPC0gUmF3QUlmb3JpYV9kZXRhaWxzX291dHB1dCAlPiUKICBmaWx0ZXIoYENsYXNzIGxhYmVsYCA9PSAnVGlzc3VlJykgJT4lCiAgZ3JvdXBfYnkoUk9JKSAlPiUKICBzdW1tYXJpc2Uocm9pX3Rpc3N1ZV9hcmVhX3VtMiA9IHN1bShgQXJlYSAozrxtwrIpYCkpCgojIEFyZWEgb2YgcGFyZW50IHRpc3N1ZQpwYXJlbnRfdGlzc3VlX2FyZWFzIDwtIFJhd0FJZm9yaWFfZGV0YWlsc19vdXRwdXQgJT4lCiAgZmlsdGVyKGBDbGFzcyBsYWJlbGAgPT0gJ1Rpc3N1ZScpICU+JQogIGdyb3VwX2J5KGBBcmVhL29iamVjdCBJRGApICU+JQogIHN1bW1hcmlzZShwYXJlbnRfdGlzc3VlX2FyZWFfdW0yID0gc3VtKGBBcmVhICjOvG3CsilgKSkKcGFyZW50X3Rpc3N1ZV9hcmVhcyRwYXJlbnRfdGlzc3VlX2FyZWFfbW0yIDwtIHBhcmVudF90aXNzdWVfYXJlYXMkcGFyZW50X3Rpc3N1ZV9hcmVhX3VtMiAqIDAuMDAwMDAxCgpyb2lfbGV0dGVyX3BhdHRlcm4gPC0gUk9JS2V5JGBsZXR0ZXJzIGZvdW5kIGluIFJPSSBsYWJlbCB1c2VkIHRvIGNvZGUgYnJhaW4gcmVnaW9uYCAlPiUgcGFzdGUoY29sbGFwc2UgPSAnfCcpCgojIENvbWJpbmUgdHJlYXRtZW50IGluZm8gd2l0aCBkYXRhClJhd0FJZm9yaWFfZGV0YWlsc19vdXRwdXRfUGx1c1RyZWF0bWVudEluZm8gPC0gUmF3QUlmb3JpYV9kZXRhaWxzX291dHB1dCAlPiUKICBtdXRhdGUoCiAgICBhcmVhX3RvX2NpcmN1bWZlcmVuY2UgPSBgQXJlYSAozrxtwrIpYCAvIGBDaXJjdW1mZXJlbmNlICjCtW0pYAogICkgJT4lCiAgbGVmdF9qb2luKAogICAgcm9pX3Rpc3N1ZV9hcmVhcywKICAgIGJ5ID0gJ1JPSScKICApICU+JQogIGxlZnRfam9pbigKICAgIHBhcmVudF90aXNzdWVfYXJlYXMsCiAgICBieSA9IGMoJ1BhcmVudCBhcmVhIElEJyA9ICdBcmVhL29iamVjdCBJRCcpCiAgKSAlPiUKICAjIEFkZCBicmFpbiByZWdpb24gd2l0aCByb2kgbGFiZWwga2V5CiAgbXV0YXRlKAogICAgcm9pX2xldHRlcnMgPSByZWdleHByKHJvaV9sZXR0ZXJfcGF0dGVybiwgUk9JKSAlPiUgcmVnbWF0Y2hlcyhST0ksIC4pCiAgKSAlPiUKICBsZWZ0X2pvaW4oCiAgICBzZWxlY3QoUk9JS2V5LCBgbGV0dGVycyBmb3VuZCBpbiBST0kgbGFiZWwgdXNlZCB0byBjb2RlIGJyYWluIHJlZ2lvbmAsIGBCcmFpbiByZWdpb25gKSwKICAgIGJ5ID0gYygncm9pX2xldHRlcnMnID0gJ2xldHRlcnMgZm91bmQgaW4gUk9JIGxhYmVsIHVzZWQgdG8gY29kZSBicmFpbiByZWdpb24nKQogICkgJT4lCiAgIyBBZGQgdHJlYXRtZW50IGluZm8gd2l0aCBpbWFnZSBuYW1lCiAgbGVmdF9qb2luKAogICAgVHJlYXRtZW50SW5mb0tleSwKICAgIGJ5ID0gYygnSW1hZ2UnID0gJ2ltYWdlIG5hbWUnKQogICkKCiNzdGFydCBwdElJClN1bW1hcnlCeVJPSSA8LSBSYXdBSWZvcmlhX2RldGFpbHNfb3V0cHV0X1BsdXNUcmVhdG1lbnRJbmZvICU+JQogICNmaWx0ZXIoYENsYXNzIGxhYmVsYCAhPSAnVGlzc3VlJykgJT4lCiAgZ3JvdXBfYnkoSW1hZ2UsIFJPSSwgYENsYXNzIGxhYmVsYCwgYEJyYWluIHJlZ2lvbmAsIGB0cmVhdG1lbnQgaW5mb2AsIGBwYXJlbnRfdGlzc3VlX2FyZWFfbW0yYCApICU+JQogIG11dGF0ZShDb3VudCA9IG4oKSkgJT4lCiAgI2ZpbHRlcihDb3VudCA+MTAwKSAlPiUgI3RoaXMgaXMgYSBsaXR0bGUgYXJiaXRyYXJ5IGJ1dCByZW1vdmVzIGZhaWxlZCBST0lzCiAgZ3JvdXBfYnkoSW1hZ2UsIFJPSSwgYENsYXNzIGxhYmVsYCwgYEJyYWluIHJlZ2lvbmAsIGB0cmVhdG1lbnQgaW5mb2AsIGBwYXJlbnRfdGlzc3VlX2FyZWFfbW0yYCwgYENvdW50YCApICU+JQogIAogIHN1bW1hcmlzZSggCiAgICBtZWFuX29iamVjdF9kaWFtZXRlcl91bSA9IG1lYW4oYE9iamVjdCBkaWFtZXRlciAozrxtKWAsIG5hLnJtID0gVFJVRSksCiAgICBtZWFuX2FyZWFfdG9fY2lyY3VtZmVyZW5jZSA9IG1lYW4oYGFyZWFfdG9fY2lyY3VtZmVyZW5jZWAsIG5hLnJtID0gVFJVRSksCiAgICBtZWFuX2FyZWFfdW0gPSBtZWFuKGBBcmVhICjOvG3CsilgLCBuYS5ybSA9IFRSVUUpLAogICAgbWVhbl9jaXJjdW1mZXJlbmNlX3VtID0gbWVhbihgQ2lyY3VtZmVyZW5jZSAowrVtKWAsIG5hLnJtID0gVFJVRSksCiAgICBtZWFuX2xlbmd0aF91bSA9IG1lYW4oYExlbmd0aCAowrVtKWAsIG5hLnJtID0gVFJVRSksCiAgICBtZWFuX2IgPSBtZWFuKGBCYCwgbmEucm0gPSBUUlVFKSwKICAgIG1lYW5fZyA9IG1lYW4oYEdgLCBuYS5ybSA9IFRSVUUpLAogICAgbWVhbl9yID0gbWVhbihgUmAsIG5hLnJtID0gVFJVRSksCiAgKQoKb3Blbnhsc3g6OndyaXRlLnhsc3goU3VtbWFyeUJ5Uk9JLAogICAgICAgICAgICAgICAgICAgICBmaWxlID0gIlN1bW1hcnlCeVJPSS54bHN4IikKI3B0LklJSQojQnJhaW4gcmVnaW9uMQpsaWJyYXJ5KHN2RGlhbG9ncykKQnJhaW5SZWdpb24xX3VzZXIuaW5wdXQgPC0gZGxnSW5wdXQoIkVudGVyIHRoZSBsYWJlbCB1c2VkIGZvciBicmFpbiByZWdpb24gMSIsIFN5cy5pbmZvKClbInVzZXIiXSkkcmVzCgpCUjEgPC0gU3VtbWFyeUJ5Uk9JICU+JSAKICBmaWx0ZXIoYEJyYWluIHJlZ2lvbmAgJWluJSBjKEJyYWluUmVnaW9uMV91c2VyLmlucHV0KSklPiUKICBncm91cF9ieShJbWFnZSwgYHRyZWF0bWVudCBpbmZvYCwgICkgJT4lCiAgCiAgc3VtbWFyaXNlKCAKICAgIHRvdGFsX0NvdW50ID0gc3VtKENvdW50LCBuYS5ybSA9IFRSVUUpLAogICAgdG90YWxfUGFyZW50X1Rpc3N1ZV9BcmVhX21tMiA9IHN1bShgcGFyZW50X3Rpc3N1ZV9hcmVhX21tMmAsIG5hLnJtID0gVFJVRSksCiAgICBtZWFuX1BhcmVudF9UaXNzdWVfQXJlYV9tbTIgPSBtZWFuKGBwYXJlbnRfdGlzc3VlX2FyZWFfbW0yYCwgbmEucm0gPSBUUlVFKSwKICAgIG1lYW5fT2JqZWN0X0RpYW1ldGVyX3VtID0gbWVhbihgbWVhbl9vYmplY3RfZGlhbWV0ZXJfdW1gLCBuYS5ybSA9IFRSVUUpLAogICAgbWVhbl9BcmVhX3RvX0NpcmN1bWZlcmVuY2UgPSBtZWFuKGBtZWFuX2FyZWFfdG9fY2lyY3VtZmVyZW5jZWAsIG5hLnJtID0gVFJVRSksCiAgICBtZWFuX0FyZWFfdW0gPSBtZWFuKGBtZWFuX2FyZWFfdW1gLCBuYS5ybSA9IFRSVUUpLAogICAgbWVhbl9DaXJjdW1mZXJlbmNlX3VtID0gbWVhbihgbWVhbl9jaXJjdW1mZXJlbmNlX3VtYCwgbmEucm0gPSBUUlVFKSwKICAgIG1lYW5fTGVuZ3RoX3VtID0gbWVhbihgbWVhbl9sZW5ndGhfdW1gLCBuYS5ybSA9IFRSVUUpLAogICAgbWVhbl9CID0gbWVhbihgbWVhbl9iYCwgbmEucm0gPSBUUlVFKSwKICAgIG1lYW5fRyA9IG1lYW4oYG1lYW5fZ2AsIG5hLnJtID0gVFJVRSksCiAgICBtZWFuX1IgPSBtZWFuKGBtZWFuX3JgLCBuYS5ybSA9IFRSVUUpLAogICkKCm9wZW54bHN4Ojp3cml0ZS54bHN4KEJSMSwKICAgICAgICAgICAgICAgICAgICAgZmlsZSA9IHBhc3RlMChCcmFpblJlZ2lvbjFfdXNlci5pbnB1dCwnLnhsc3gnKSkKCiNCcmFpbiByZWdpb24yCmxpYnJhcnkoc3ZEaWFsb2dzKQpCcmFpblJlZ2lvbjJfdXNlci5pbnB1dCA8LSBkbGdJbnB1dCgiRW50ZXIgdGhlIGxhYmVsIHVzZWQgZm9yIGJyYWluIHJlZ2lvbiAyIiwgU3lzLmluZm8oKVsidXNlciJdKSRyZXMKCkJSMiA8LSBTdW1tYXJ5QnlST0kgJT4lIAogIGZpbHRlcihgQnJhaW4gcmVnaW9uYCAlaW4lIGMoQnJhaW5SZWdpb24yX3VzZXIuaW5wdXQpKSU+JQogIGdyb3VwX2J5KEltYWdlLCBgdHJlYXRtZW50IGluZm9gLCAgKSAlPiUKICAKICBzdW1tYXJpc2UoIAogICAgdG90YWxfQ291bnQgPSBzdW0oQ291bnQsIG5hLnJtID0gVFJVRSksCiAgICB0b3RhbF9QYXJlbnRfVGlzc3VlX0FyZWFfbW0yID0gc3VtKGBwYXJlbnRfdGlzc3VlX2FyZWFfbW0yYCwgbmEucm0gPSBUUlVFKSwKICAgIG1lYW5fUGFyZW50X1Rpc3N1ZV9BcmVhX21tMiA9IG1lYW4oYHBhcmVudF90aXNzdWVfYXJlYV9tbTJgLCBuYS5ybSA9IFRSVUUpLAogICAgbWVhbl9PYmplY3RfRGlhbWV0ZXJfdW0gPSBtZWFuKGBtZWFuX29iamVjdF9kaWFtZXRlcl91bWAsIG5hLnJtID0gVFJVRSksCiAgICBtZWFuX0FyZWFfdG9fQ2lyY3VtZmVyZW5jZSA9IG1lYW4oYG1lYW5fYXJlYV90b19jaXJjdW1mZXJlbmNlYCwgbmEucm0gPSBUUlVFKSwKICAgIG1lYW5fQXJlYV91bSA9IG1lYW4oYG1lYW5fYXJlYV91bWAsIG5hLnJtID0gVFJVRSksCiAgICBtZWFuX0NpcmN1bWZlcmVuY2VfdW0gPSBtZWFuKGBtZWFuX2NpcmN1bWZlcmVuY2VfdW1gLCBuYS5ybSA9IFRSVUUpLAogICAgbWVhbl9MZW5ndGhfdW0gPSBtZWFuKGBtZWFuX2xlbmd0aF91bWAsIG5hLnJtID0gVFJVRSksCiAgICBtZWFuX0IgPSBtZWFuKGBtZWFuX2JgLCBuYS5ybSA9IFRSVUUpLAogICAgbWVhbl9HID0gbWVhbihgbWVhbl9nYCwgbmEucm0gPSBUUlVFKSwKICAgIG1lYW5fUiA9IG1lYW4oYG1lYW5fcmAsIG5hLnJtID0gVFJVRSksCiAgKQoKb3Blbnhsc3g6OndyaXRlLnhsc3goQlIyLAogICAgICAgICAgICAgICAgICAgICBmaWxlID0gcGFzdGUwKEJyYWluUmVnaW9uMl91c2VyLmlucHV0LCcueGxzeCcpKQoKI0JyYWluIHJlZ2lvbjMKbGlicmFyeShzdkRpYWxvZ3MpCkJyYWluUmVnaW9uM191c2VyLmlucHV0IDwtIGRsZ0lucHV0KCJFbnRlciB0aGUgbGFiZWwgdXNlZCBmb3IgYnJhaW4gcmVnaW9uIDMiLCBTeXMuaW5mbygpWyJ1c2VyIl0pJHJlcwoKQlIzIDwtIFN1bW1hcnlCeVJPSSAlPiUgCiAgZmlsdGVyKGBCcmFpbiByZWdpb25gICVpbiUgYyhCcmFpblJlZ2lvbjNfdXNlci5pbnB1dCkpJT4lCiAgZ3JvdXBfYnkoSW1hZ2UsIGB0cmVhdG1lbnQgaW5mb2AsICApICU+JQogIAogIHN1bW1hcmlzZSggCiAgICB0b3RhbF9Db3VudCA9IHN1bShDb3VudCwgbmEucm0gPSBUUlVFKSwKICAgIHRvdGFsX1BhcmVudF9UaXNzdWVfQXJlYV9tbTIgPSBzdW0oYHBhcmVudF90aXNzdWVfYXJlYV9tbTJgLCBuYS5ybSA9IFRSVUUpLAogICAgbWVhbl9QYXJlbnRfVGlzc3VlX0FyZWFfbW0yID0gbWVhbihgcGFyZW50X3Rpc3N1ZV9hcmVhX21tMmAsIG5hLnJtID0gVFJVRSksCiAgICBtZWFuX09iamVjdF9EaWFtZXRlcl91bSA9IG1lYW4oYG1lYW5fb2JqZWN0X2RpYW1ldGVyX3VtYCwgbmEucm0gPSBUUlVFKSwKICAgIG1lYW5fQXJlYV90b19DaXJjdW1mZXJlbmNlID0gbWVhbihgbWVhbl9hcmVhX3RvX2NpcmN1bWZlcmVuY2VgLCBuYS5ybSA9IFRSVUUpLAogICAgbWVhbl9BcmVhX3VtID0gbWVhbihgbWVhbl9hcmVhX3VtYCwgbmEucm0gPSBUUlVFKSwKICAgIG1lYW5fQ2lyY3VtZmVyZW5jZV91bSA9IG1lYW4oYG1lYW5fY2lyY3VtZmVyZW5jZV91bWAsIG5hLnJtID0gVFJVRSksCiAgICBtZWFuX0xlbmd0aF91bSA9IG1lYW4oYG1lYW5fbGVuZ3RoX3VtYCwgbmEucm0gPSBUUlVFKSwKICAgIG1lYW5fQiA9IG1lYW4oYG1lYW5fYmAsIG5hLnJtID0gVFJVRSksCiAgICBtZWFuX0cgPSBtZWFuKGBtZWFuX2dgLCBuYS5ybSA9IFRSVUUpLAogICAgbWVhbl9SID0gbWVhbihgbWVhbl9yYCwgbmEucm0gPSBUUlVFKSwKICApCgpvcGVueGxzeDo6d3JpdGUueGxzeChCUjMsCiAgICAgICAgICAgICAgICAgICAgIGZpbGUgPSBwYXN0ZTAoQnJhaW5SZWdpb24zX3VzZXIuaW5wdXQsJy54bHN4JykpCgojQnJhaW4gcmVnaW9uNApsaWJyYXJ5KHN2RGlhbG9ncykKQnJhaW5SZWdpb240X3VzZXIuaW5wdXQgPC0gZGxnSW5wdXQoIkVudGVyIHRoZSBsYWJlbCB1c2VkIGZvciBicmFpbiByZWdpb24gNCIsIFN5cy5pbmZvKClbInVzZXIiXSkkcmVzCgpCUjQgPC0gU3VtbWFyeUJ5Uk9JICU+JSAKICBmaWx0ZXIoYEJyYWluIHJlZ2lvbmAgJWluJSBjKEJyYWluUmVnaW9uNF91c2VyLmlucHV0KSklPiUKICBncm91cF9ieShJbWFnZSwgYHRyZWF0bWVudCBpbmZvYCwgICkgJT4lCiAgCiAgc3VtbWFyaXNlKCAKICAgIHRvdGFsX0NvdW50ID0gc3VtKENvdW50LCBuYS5ybSA9IFRSVUUpLAogICAgdG90YWxfUGFyZW50X1Rpc3N1ZV9BcmVhX21tMiA9IHN1bShgcGFyZW50X3Rpc3N1ZV9hcmVhX21tMmAsIG5hLnJtID0gVFJVRSksCiAgICBtZWFuX1BhcmVudF9UaXNzdWVfQXJlYV9tbTIgPSBtZWFuKGBwYXJlbnRfdGlzc3VlX2FyZWFfbW0yYCwgbmEucm0gPSBUUlVFKSwKICAgIG1lYW5fT2JqZWN0X0RpYW1ldGVyX3VtID0gbWVhbihgbWVhbl9vYmplY3RfZGlhbWV0ZXJfdW1gLCBuYS5ybSA9IFRSVUUpLAogICAgbWVhbl9BcmVhX3RvX0NpcmN1bWZlcmVuY2UgPSBtZWFuKGBtZWFuX2FyZWFfdG9fY2lyY3VtZmVyZW5jZWAsIG5hLnJtID0gVFJVRSksCiAgICBtZWFuX0FyZWFfdW0gPSBtZWFuKGBtZWFuX2FyZWFfdW1gLCBuYS5ybSA9IFRSVUUpLAogICAgbWVhbl9DaXJjdW1mZXJlbmNlX3VtID0gbWVhbihgbWVhbl9jaXJjdW1mZXJlbmNlX3VtYCwgbmEucm0gPSBUUlVFKSwKICAgIG1lYW5fTGVuZ3RoX3VtID0gbWVhbihgbWVhbl9sZW5ndGhfdW1gLCBuYS5ybSA9IFRSVUUpLAogICAgbWVhbl9CID0gbWVhbihgbWVhbl9iYCwgbmEucm0gPSBUUlVFKSwKICAgIG1lYW5fRyA9IG1lYW4oYG1lYW5fZ2AsIG5hLnJtID0gVFJVRSksCiAgICBtZWFuX1IgPSBtZWFuKGBtZWFuX3JgLCBuYS5ybSA9IFRSVUUpLAogICkKCm9wZW54bHN4Ojp3cml0ZS54bHN4KEJSNCwKICAgICAgICAgICAgICAgICAgICAgZmlsZSA9IHBhc3RlMChCcmFpblJlZ2lvbjRfdXNlci5pbnB1dCwnLnhsc3gnKSkKCiNCcmFpbiByZWdpb241CmxpYnJhcnkoc3ZEaWFsb2dzKQpCcmFpblJlZ2lvbjVfdXNlci5pbnB1dCA8LSBkbGdJbnB1dCgiRW50ZXIgdGhlIGxhYmVsIHVzZWQgZm9yIGJyYWluIHJlZ2lvbiA1IiwgU3lzLmluZm8oKVsidXNlciJdKSRyZXMKCkJSNSA8LSBTdW1tYXJ5QnlST0kgJT4lIAogIGZpbHRlcihgQnJhaW4gcmVnaW9uYCAlaW4lIGMoQnJhaW5SZWdpb241X3VzZXIuaW5wdXQpKSU+JQogIGdyb3VwX2J5KEltYWdlLCBgdHJlYXRtZW50IGluZm9gLCAgKSAlPiUKICAKICBzdW1tYXJpc2UoIAogICAgdG90YWxfQ291bnQgPSBzdW0oQ291bnQsIG5hLnJtID0gVFJVRSksCiAgICB0b3RhbF9QYXJlbnRfVGlzc3VlX0FyZWFfbW0yID0gc3VtKGBwYXJlbnRfdGlzc3VlX2FyZWFfbW0yYCwgbmEucm0gPSBUUlVFKSwKICAgIG1lYW5fUGFyZW50X1Rpc3N1ZV9BcmVhX21tMiA9IG1lYW4oYHBhcmVudF90aXNzdWVfYXJlYV9tbTJgLCBuYS5ybSA9IFRSVUUpLAogICAgbWVhbl9PYmplY3RfRGlhbWV0ZXJfdW0gPSBtZWFuKGBtZWFuX29iamVjdF9kaWFtZXRlcl91bWAsIG5hLnJtID0gVFJVRSksCiAgICBtZWFuX0FyZWFfdG9fQ2lyY3VtZmVyZW5jZSA9IG1lYW4oYG1lYW5fYXJlYV90b19jaXJjdW1mZXJlbmNlYCwgbmEucm0gPSBUUlVFKSwKICAgIG1lYW5fQXJlYV91bSA9IG1lYW4oYG1lYW5fYXJlYV91bWAsIG5hLnJtID0gVFJVRSksCiAgICBtZWFuX0NpcmN1bWZlcmVuY2VfdW0gPSBtZWFuKGBtZWFuX2NpcmN1bWZlcmVuY2VfdW1gLCBuYS5ybSA9IFRSVUUpLAogICAgbWVhbl9MZW5ndGhfdW0gPSBtZWFuKGBtZWFuX2xlbmd0aF91bWAsIG5hLnJtID0gVFJVRSksCiAgICBtZWFuX0IgPSBtZWFuKGBtZWFuX2JgLCBuYS5ybSA9IFRSVUUpLAogICAgbWVhbl9HID0gbWVhbihgbWVhbl9nYCwgbmEucm0gPSBUUlVFKSwKICAgIG1lYW5fUiA9IG1lYW4oYG1lYW5fcmAsIG5hLnJtID0gVFJVRSksCiAgKQoKb3Blbnhsc3g6OndyaXRlLnhsc3goQlI1LAogICAgICAgICAgICAgICAgICAgICBmaWxlID0gcGFzdGUwKEJyYWluUmVnaW9uNV91c2VyLmlucHV0LCcueGxzeCcpKQoKI0JyYWluIHJlZ2lvbjYKbGlicmFyeShzdkRpYWxvZ3MpCkJyYWluUmVnaW9uNl91c2VyLmlucHV0IDwtIGRsZ0lucHV0KCJFbnRlciB0aGUgbGFiZWwgdXNlZCBmb3IgYnJhaW4gcmVnaW9uIDYiLCBTeXMuaW5mbygpWyJ1c2VyIl0pJHJlcwoKQlI2IDwtIFN1bW1hcnlCeVJPSSAlPiUgCiAgZmlsdGVyKGBCcmFpbiByZWdpb25gICVpbiUgYyhCcmFpblJlZ2lvbjZfdXNlci5pbnB1dCkpJT4lCiAgZ3JvdXBfYnkoSW1hZ2UsIGB0cmVhdG1lbnQgaW5mb2AsICApICU+JQogIAogIHN1bW1hcmlzZSggCiAgICB0b3RhbF9Db3VudCA9IHN1bShDb3VudCwgbmEucm0gPSBUUlVFKSwKICAgIHRvdGFsX1BhcmVudF9UaXNzdWVfQXJlYV9tbTIgPSBzdW0oYHBhcmVudF90aXNzdWVfYXJlYV9tbTJgLCBuYS5ybSA9IFRSVUUpLAogICAgbWVhbl9QYXJlbnRfVGlzc3VlX0FyZWFfbW0yID0gbWVhbihgcGFyZW50X3Rpc3N1ZV9hcmVhX21tMmAsIG5hLnJtID0gVFJVRSksCiAgICBtZWFuX09iamVjdF9EaWFtZXRlcl91bSA9IG1lYW4oYG1lYW5fb2JqZWN0X2RpYW1ldGVyX3VtYCwgbmEucm0gPSBUUlVFKSwKICAgIG1lYW5fQXJlYV90b19DaXJjdW1mZXJlbmNlID0gbWVhbihgbWVhbl9hcmVhX3RvX2NpcmN1bWZlcmVuY2VgLCBuYS5ybSA9IFRSVUUpLAogICAgbWVhbl9BcmVhX3VtID0gbWVhbihgbWVhbl9hcmVhX3VtYCwgbmEucm0gPSBUUlVFKSwKICAgIG1lYW5fQ2lyY3VtZmVyZW5jZV91bSA9IG1lYW4oYG1lYW5fY2lyY3VtZmVyZW5jZV91bWAsIG5hLnJtID0gVFJVRSksCiAgICBtZWFuX0xlbmd0aF91bSA9IG1lYW4oYG1lYW5fbGVuZ3RoX3VtYCwgbmEucm0gPSBUUlVFKSwKICAgIG1lYW5fQiA9IG1lYW4oYG1lYW5fYmAsIG5hLnJtID0gVFJVRSksCiAgICBtZWFuX0cgPSBtZWFuKGBtZWFuX2dgLCBuYS5ybSA9IFRSVUUpLAogICAgbWVhbl9SID0gbWVhbihgbWVhbl9yYCwgbmEucm0gPSBUUlVFKSwKICApCgpvcGVueGxzeDo6d3JpdGUueGxzeChCUjYsCiAgICAgICAgICAgICAgICAgICAgIGZpbGUgPSBwYXN0ZTAoQnJhaW5SZWdpb242X3VzZXIuaW5wdXQsJy54bHN4JykpCgojQnJhaW4gcmVnaW9uNwpsaWJyYXJ5KHN2RGlhbG9ncykKQnJhaW5SZWdpb243X3VzZXIuaW5wdXQgPC0gZGxnSW5wdXQoIkVudGVyIHRoZSBsYWJlbCB1c2VkIGZvciBicmFpbiByZWdpb24gNyIsIFN5cy5pbmZvKClbInVzZXIiXSkkcmVzCgpCUjcgPC0gU3VtbWFyeUJ5Uk9JICU+JSAKICBmaWx0ZXIoYEJyYWluIHJlZ2lvbmAgJWluJSBjKEJyYWluUmVnaW9uN191c2VyLmlucHV0KSklPiUKICBncm91cF9ieShJbWFnZSwgYHRyZWF0bWVudCBpbmZvYCwgICkgJT4lCiAgCiAgc3VtbWFyaXNlKCAKICAgIHRvdGFsX0NvdW50ID0gc3VtKENvdW50LCBuYS5ybSA9IFRSVUUpLAogICAgdG90YWxfUGFyZW50X1Rpc3N1ZV9BcmVhX21tMiA9IHN1bShgcGFyZW50X3Rpc3N1ZV9hcmVhX21tMmAsIG5hLnJtID0gVFJVRSksCiAgICBtZWFuX1BhcmVudF9UaXNzdWVfQXJlYV9tbTIgPSBtZWFuKGBwYXJlbnRfdGlzc3VlX2FyZWFfbW0yYCwgbmEucm0gPSBUUlVFKSwKICAgIG1lYW5fT2JqZWN0X0RpYW1ldGVyX3VtID0gbWVhbihgbWVhbl9vYmplY3RfZGlhbWV0ZXJfdW1gLCBuYS5ybSA9IFRSVUUpLAogICAgbWVhbl9BcmVhX3RvX0NpcmN1bWZlcmVuY2UgPSBtZWFuKGBtZWFuX2FyZWFfdG9fY2lyY3VtZmVyZW5jZWAsIG5hLnJtID0gVFJVRSksCiAgICBtZWFuX0FyZWFfdW0gPSBtZWFuKGBtZWFuX2FyZWFfdW1gLCBuYS5ybSA9IFRSVUUpLAogICAgbWVhbl9DaXJjdW1mZXJlbmNlX3VtID0gbWVhbihgbWVhbl9jaXJjdW1mZXJlbmNlX3VtYCwgbmEucm0gPSBUUlVFKSwKICAgIG1lYW5fTGVuZ3RoX3VtID0gbWVhbihgbWVhbl9sZW5ndGhfdW1gLCBuYS5ybSA9IFRSVUUpLAogICAgbWVhbl9CID0gbWVhbihgbWVhbl9iYCwgbmEucm0gPSBUUlVFKSwKICAgIG1lYW5fRyA9IG1lYW4oYG1lYW5fZ2AsIG5hLnJtID0gVFJVRSksCiAgICBtZWFuX1IgPSBtZWFuKGBtZWFuX3JgLCBuYS5ybSA9IFRSVUUpLAogICkKCm9wZW54bHN4Ojp3cml0ZS54bHN4KEJSNywKICAgICAgICAgICAgICAgICAgICAgZmlsZSA9IHBhc3RlMChCcmFpblJlZ2lvbjdfdXNlci5pbnB1dCwnLnhsc3gnKSkKCiNCcmFpbiByZWdpb244CmxpYnJhcnkoc3ZEaWFsb2dzKQpCcmFpblJlZ2lvbjhfdXNlci5pbnB1dCA8LSBkbGdJbnB1dCgiRW50ZXIgdGhlIGxhYmVsIHVzZWQgZm9yIGJyYWluIHJlZ2lvbiA4IiwgU3lzLmluZm8oKVsidXNlciJdKSRyZXMKCkJSOCA8LSBTdW1tYXJ5QnlST0kgJT4lIAogIGZpbHRlcihgQnJhaW4gcmVnaW9uYCAlaW4lIGMoQnJhaW5SZWdpb244X3VzZXIuaW5wdXQpKSU+JQogIGdyb3VwX2J5KEltYWdlLCBgdHJlYXRtZW50IGluZm9gLCAgKSAlPiUKICAKICBzdW1tYXJpc2UoIAogICAgdG90YWxfQ291bnQgPSBzdW0oQ291bnQsIG5hLnJtID0gVFJVRSksCiAgICB0b3RhbF9QYXJlbnRfVGlzc3VlX0FyZWFfbW0yID0gc3VtKGBwYXJlbnRfdGlzc3VlX2FyZWFfbW0yYCwgbmEucm0gPSBUUlVFKSwKICAgIG1lYW5fUGFyZW50X1Rpc3N1ZV9BcmVhX21tMiA9IG1lYW4oYHBhcmVudF90aXNzdWVfYXJlYV9tbTJgLCBuYS5ybSA9IFRSVUUpLAogICAgbWVhbl9PYmplY3RfRGlhbWV0ZXJfdW0gPSBtZWFuKGBtZWFuX29iamVjdF9kaWFtZXRlcl91bWAsIG5hLnJtID0gVFJVRSksCiAgICBtZWFuX0FyZWFfdG9fQ2lyY3VtZmVyZW5jZSA9IG1lYW4oYG1lYW5fYXJlYV90b19jaXJjdW1mZXJlbmNlYCwgbmEucm0gPSBUUlVFKSwKICAgIG1lYW5fQXJlYV91bSA9IG1lYW4oYG1lYW5fYXJlYV91bWAsIG5hLnJtID0gVFJVRSksCiAgICBtZWFuX0NpcmN1bWZlcmVuY2VfdW0gPSBtZWFuKGBtZWFuX2NpcmN1bWZlcmVuY2VfdW1gLCBuYS5ybSA9IFRSVUUpLAogICAgbWVhbl9MZW5ndGhfdW0gPSBtZWFuKGBtZWFuX2xlbmd0aF91bWAsIG5hLnJtID0gVFJVRSksCiAgICBtZWFuX0IgPSBtZWFuKGBtZWFuX2JgLCBuYS5ybSA9IFRSVUUpLAogICAgbWVhbl9HID0gbWVhbihgbWVhbl9nYCwgbmEucm0gPSBUUlVFKSwKICAgIG1lYW5fUiA9IG1lYW4oYG1lYW5fcmAsIG5hLnJtID0gVFJVRSksCiAgKQoKb3Blbnhsc3g6OndyaXRlLnhsc3goQlI4LAogICAgICAgICAgICAgICAgICAgICBmaWxlID0gcGFzdGUwKEJyYWluUmVnaW9uOF91c2VyLmlucHV0LCcueGxzeCcpKQpgYGAKCmBgYHtyfQpST0lLZXkgPC0gcmVhZHhsOjpyZWFkX3hsc3goZmlsZS5jaG9vc2UoKSkKYGBgICAgICAgICAgIAo=
